# Combined Optokinetic Treatment and Vestibular Rehabilitation to Reduce Visually Induced Dizziness in a Professional Ice Hockey Player After Concussion: A Clinical Case

**DOI:** 10.3389/fneur.2019.01200

**Published:** 2019-11-29

**Authors:** Viviana Mucci, Cornelia Meier, Mario Bizzini, Fausto Romano, Daniel Agostino, Alessandra Ventura, Giovanni Bertolini, Nina Feddermann-Demont

**Affiliations:** ^1^Swiss Concussion Center, Schulthess Clinic, Zurich, Switzerland; ^2^Department of Neurology, University Hospital and University of Zurich, Zurich, Switzerland; ^3^Human Performance Lab, Schulthess Clinic, Zurich, Switzerland

**Keywords:** concussion, ice hockey, optokinetic, dizziness, visually induced dizziness, vestibular rehabilitation, sport-related concussion

## Abstract

**Background:** The appropriate detection and therapy of concussion symptoms are of great importance to avoid long-term impairment and absence from pre-concussive activities, such as sport, school or work. Post-traumatic headache and dizziness are known as risk factors of persistent symptoms after a concussion. Dizziness has even been classified as a predictor for symptom persistence. One type of dizziness, which has never been considered is visually induced dizziness (VID) often develops as a consequence of vestibular impairment. This manuscript presents the clinical case of a 25-year-old male, professional ice hockey player, whereby a therapeutic approach to VID after concussion is demonstrated.

**Case:** A detailed interdisciplinary clinical and laboratory-assisted neurological, neurovestibular and ocular-motor examination was performed 20 days post-concussion, which indicated VID symptoms. Thus, the player qualified for a 5-day combined vestibular, balance and optokinetic therapy, which aimed to reduce the player's increased sensitivity to visual information. Each treatment day consisted of two sessions: vestibular/ocular-motor training and exposure to optokinetic stimuli combined with postural control exercises. The optokinetic stimulus was delivered in the form of a rotating disk. VID symptoms were recorded daily via posturography and a visual analog scale prior to the optokinetic sessions. The player improved over the course of each treatment day and was able to return to ice hockey 15 days after the final treatment session. Three months later the player reported no symptoms in the follow up questionnaire.

**Conclusion:** The combination of vestibular, balance and optokinetic therapy led to remission of VID symptoms in a professional ice hockey player after multiple concussions, within a short time frame after his last concussion. Thus, this case study highlights the significant benefit of treating post-concussive VID symptoms utilizing a multi-modal approach.

## Introduction to Sport-Related Concussion and Visually Induced Dizziness

Sport-related concussion (SRC) is defined as a traumatic brain injury induced by biomechanical forces (1, 2) and is currently a clinical diagnosis. SRC can lead to a variety of debilitating symptoms (3), such as headache, dizziness [prevalence of 40–60% in non-hospitalized patients following head trauma (4)], vertigo and balance problems, which are the most common symptoms reported after a SRC (5), possibly as a result of a concomitant peripheral vestibular disorder (as e.g., post-traumatic paroxysmal positional vertigo) (6), a deficit in central vestibular processing pathways (5) or both. Symptoms may persist beyond the generally accepted time frame of recovery (2 weeks) (7). In addition to this, the natural recovery time is hard to define (8–13). Dizziness has been identified as a predictor for persistent symptoms (14) and consequently, early assessment and symptom attribution is essential for defining the most appropriate therapeutic approach.

In the last few years the approach for the return to sport protocols has changed (11, 12, 15). Previously, affected athletes were recommended to rest until symptoms had resolved. Current guidelines and position papers recommend that after a brief period of rest (24–48 h), symptom-limited activity should be initiated (11). This includes an early re-start of activity gradually increasing in intensity (16), in a multi-modal approach (including cervical, sensorimotor, and vestibulo-ocular motor aspects) (16).

Despite dizziness being recognized as a frequent symptom after concussion, some specific entities of dizziness, such as *Visually Induced Dizziness (VID*) in adult athletes, have hardly been described in the literature. Given that approximately half of the brain circuits are involved in vision and eye movement control (17), SRC frequently results in visual symptoms and ocular-motor dysfunction (17). VID has been described as a condition, where dizziness can be triggered by visual stimuli such as computer screens, optic flow, repetitive visual patterns, walking in supermarket aisles and visual motion in general (18). Disorientation and dizziness may also occur when a person's visual field is overwhelmed (e.g., repetitive patterns) or due to the lack of point of reference/fixation (e.g., in intense darkness, wide open spaces) (19), or by being passenger in a car, due to the numerous stimuli moving at high speed (20, 21). This condition has been named with different terms over the years (19, 21, 22). However, since 2009, the Barany Society adopted the term VID to describe these symptoms and recently included them under the category of Persistent Postural-Perceptual Dizziness (PPPD) (23). Some patients also report visually induced vertigo on the top of VID symptoms, which indicates a type of vertigo triggered by complex patterns, distorted large visual fields or moving visual stimuli (24). It is important to distinguish VID from Visually Induced Motion Sickness (VIMS), which has been defined as a sensation of motion sickness that appears under visual stimulation such as driving simulators, movie theaters or video games (25). Outside of the sports context, VID has often been described as a consequence of different vestibular disorders, with several studies reporting the management of VID symptoms after vestibular neuritis, vestibular migraine and peripheral vestibular insults (18, 20, 21, 26). Treatments able to reduce VID are still limited (8). Vestibular rehabilitation (VR) and in particular a combination of VR with optokinetic stimuli [repetitive, progressive exposure to optokinetic stimulation (OKN)] has been proven to have the ability to decrease the effect of disorienting visual stimuli in VID patients; with an improvement in postural stability and symptoms perception (18, 27–29). Schneider et al. (30) reported favorable results with the use of cervical and VR in order to induce central compensational mechanisms in athletes affected by SRC. Currently, guidelines on the use of OKN approaches and VR in concussed athletes with VID do not exist (16). VID in an adult patient with concussion has been reported before anecdotally, but no clinical management example was found.

Ice hockey is a team contact sport known for its aggressive and fast moving actions, where players achieve a significant speed on the ice (up to 30 km per hour) (31, 32). With 1.8 concussions per 1,000 games player-hours (2), Men's ice hockey is a high risk sport for concussion (31, 33–35). Additionally, the busy and tight match schedule in ice-hockey (up to 3 matches per week) generates pressure on performance and can lead to premature return to match play, while players are still dealing with symptoms (31). Thus, this clinical case aims to present how the use of OKN treatment and VR supported the reduction of post-traumatic VID symptoms in a professional ice hockey player.

## Case Presentation

A 25 years old, male, professional ice hockey player with a history of 3 concussions (2015, 2017, 2018) was assessed on December 3rd, 2018 (20 days after the last head trauma). A written information & consent form was provided to the player upon his arrival. He was explained in details about the diagnostic procedure of evaluation for concussion as well as the therapeutic approaches. He was informed that he could stop the intervention at any time without any particular reasons or without damaging his relationship with his doctor and therapists. Risk, benefits as well as the use of his data in an anonymous form were described and explained to the player. The player provided the permission to use his data for the purpose of this clinical case.

### History

Initial symptoms immediately after the last hit were blurred vision fogginess, fatigue, nausea and sensitivity to light. After an initial period of 7 days of rest he started the RTS protocol but had to stop on the level of non-contact training on the ice [between level 3 and 4—RTS of McCrory et al. (36)].

### Symptoms (as Reported During Consultation)

On the first day of assessment the predominant problems were attributed to visual and vestibular domains (blurred vision, fogginess, light-headedness, drowsiness, including nausea). With the information provided by the patient, it was not possible to exclude that the reported symptoms were pre-existing, residual symptoms from the previous concussions. Current complains resembled his symptoms during the previous concussions. Retrospectively the player admitted, that he never had full symptom relieve (as e.g., prefers sun-glasses due to sensitivity to light) and tried to avoid certain situations (e.g., shopping in a supermarket), since the previous trauma occurred (2nd concussion).

### Neurological Examinations

During the clinical neurological examination impairments were identified in postural stability in the following conditions: with eyes closed, during ocular-motor tasks (convergence) and when exposed to OKN stimulation. Positioning maneuvers were performed for all six semi-circular canals without nystagmus/hints of Benign Paroxysmal Positional Vertigo (BPPV). However, the player was successfully treated for BPPV after his previous concussion. After the first concussion, the patient performed a structural magnetic resonance imaging (MRI), which was recommended by his team physician and resulted in no abnormalities. No specific imaging examinations (e.g., MRI spectroscopy) were performed. On the basis of the clinical neurological examination VID symptoms were identified by means of questionnaires: Situational Characteristic Questionnaire (SCQ) and specific Visual Vertigo Questionnaire using an Analog Scale (VVAS) (29).

### Laboratory Assisted Examinations

Laboratory assisted vestibular and ocular-motor tests were normal [Video head impulse test, ocular and cervical Vestibular Evoked Myogenic Potential (oVEMP-cVEMP)]. For the oVEMP the bone-conducted burst type vibration of 2 ms was used (500 Hz sinus wave). For the cVEMP both air- and bone-conducted stimulations were used. Air-conducted stimulation tone burst was performed with a type of stimulation of 6 ms, 500 Hz sinus wave. As per routine the test always initiates with the air conducted stimulation at 95 dB when reflexes reported then the stimulation decreased at 90 dB and then 70 dB. If no reflexes observed, the stimuli increased to 100 dB. Although in this case, the air-conducted cVEMPs resulted normal, the bone-conducted was also performed (burst type 6 ms- 500 Hz sinus wave), since it was evaluated after the previous concussion. All results were normal.

Vestibulo-oculography including: vestibular caloric irrigation test, and subjective visual vertical assessment. A pathological binocular cyclorotation to the left (fundus photography) was objectified. The result of the Sensory Organization Test (SOT) and the Head-Shake SOT objectified a marginal vestibular deficiency [VEST Sensory analysis score: 52% (normative value >55%); HS SOT ratios: 67%]. The gaze stability test (GST) and active dynamic visual acuity (DVA) tests were normal.

### Physiotherapeutic Examinations

During the vestibular-ocular-motor screening (VOMS) (37) the patient was slightly symptomatic (eye pressure and dizziness) following some ocular-motor (horizontal saccades) and vestibular (horizontal VOR) tasks. The visual motion sensitivity test was able to induce a high intensive dizziness and fogginess. Similar results were reported during a functional test involving rotatory movements of the head and of the whole body. Cervical spine examination was normal.

### Diagnosis

Combining the results from the different examinations, the symptoms were classified as a visual-vestibular dysfunction with reduced gaze stabilization and VID.

## The Therapy

### Therapeutic Intervention and Monitoring Methods

The therapeutic concept was based on the combination of vestibular, ocular-motor and OKN treatment. The player performed 45 min session of OKN exposure and 1 h of vestibular-ocular-motor exercises for 5 days over 2 calendar weeks. For the “resting” days, the player was instructed to perform exercises at home.

**Figure 1:**
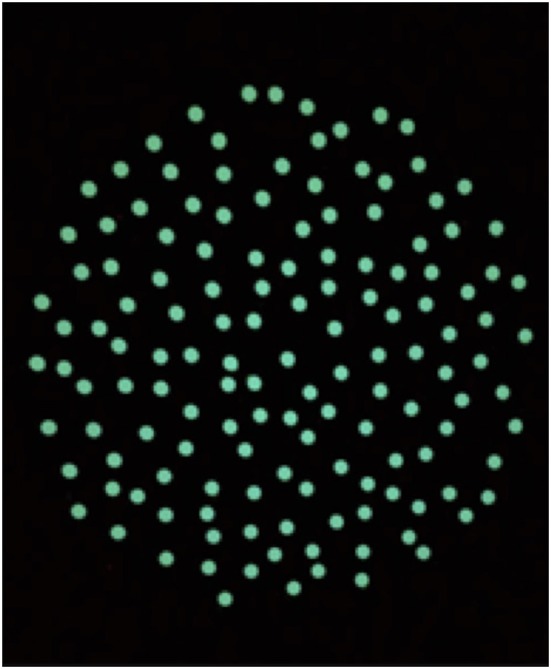
Optokinetic (OKN) rotatory disk used for inducing the OKN stimuli.

The OKN treatment was performed according to a modified version of the treatment of VID patients performed in a previous study by Pavlou et al. (29). The treatment was held in a total dark room, free from points of references. The visual stimulus consisted of a circle of dots projected on a screen (Figure 1), reproducing the appearance of the optokinetic disk (29). The player was instructed to maintain gaze on the stable dot in the center of the projected disk. The projected disk was rotated around the central, stable dot to create an OKN stimulus in the roll plane. The therapist adapted the speed of rotation, depending on the player reaction, by 5°-10°/s per session following verbal feedbacks. Multiple sessions a day were performed. The disk started at a rotatory speed of 30°/s during the first session and reached 60°/s during the last session. As the therapy progressed the player was asked to perform different tasks while staring at the OKN disk (Table 1). Every task was repeated for the same amount of time in both directions of the disk (rotation of the disk: clockwise and anticlockwise). Currently, we decided to start in a randomized order the first direction of the disk, since there is no evidence that maintaining a direction would be affecting the treatment. The VR consisted initially of ocular-motor and vestibular exercises selected on the basis of the VOMS-components that elicited symptoms. These exercises were progressively combined and integrated within complex motor tasks in the following days (Table 1).

**Table 1:** Description of the sequence between vestibular rehabilitation and optokinetic treatment performed during 2 weeks of treatment.

**Therapy day**	**Vestibular rehabilitation**	**Optokinetic training**
1	Horizontal and vertical saccadic training: 3 times for 30 s Binocular view and convergence training Vestibular exercises: gaze stability while moving the head (YAW)	Optokinetic training
2	Ocular-motor (OC) exercises: Vergence/accommodation training	Optokinetic training
Break		
Break		Behavioral task (exposure to real life stimuli)
Break	OC exercises at home (see day 2 for details)	
Break		
3	OC + VR: Gaze stability and vergence/accommodation training. Capturing a moving visual target while turning 180 degrees on a trampoline. Balance training on unstable surfaces (Foam, BOSU) with changing tasks	Optokinetic training
4	OC + VR: Gaze stability and vergence/accommodation training while walking Gaze stability and capturing a moving visual target while turning fast Gaze stability and postural orientation while moving in the transverse plane. Balance training on unstable surfaces (Foam, BOSU) with changing tasks	Optokinetic training
5	OC + VR: Gaze stability and vergence/accommodation training while walking Gaze stability and capturing a moving visual target while turning fast Gaze stability and postural orientation while moving in the transverse plane. Balance training on unstable surfaces (Foam, BOSU) with changing tasks	Optokinetic training

### Subjective Outcomes

Verbal feedbacks on symptoms and VVAS questionnaires were recorded every day prior to the OKN treatment. To assess relationship between the VVAS scores and day of treatment, the spearman's rank correlation coefficient was also used.

### Objective Outcomes

Postural stability was measured before every OKN session. The player was asked to stand upright for 1 min, with feet apart (hip width) on a Wii Balance board (Nintendo Co., Ltd.) (38), with eyes closed, and barefoot (38–41) acquiring his center of pressure (CoP). The data was acquired with a custom designed program based on the Colorado University Wii Balance Board code [Neuromechanics Laboratory at Colorado University (42)] and then analyzed using MATLAB (Release 2017b, developed by The MathWorks, Inc., Natick, Massachusetts, United States). Specially, the data was filtered using a Butterworth filter of fourth order and a cut-off frequency of 0.17 Hz (43). As output parameter, we computed from the sway path of the COP the Confidence Ellipse Area (CEA, i.e., the 95% confidence ellipse area that encloses approximately 95% of the points on the sway path) since has been widely used before (43, 44).

## Results

### Subjective Outcome

The symptoms reported by the player on the first treatment day (high intensive dizziness, postural instability and difficulties in maintaining visual acuity) improved throughout the 5 days of treatment. The player mostly mentioned to have temporal headache, which usually vanished after a few hours after the sessions, and fatigue. This aspect could be a consequence of the OKN stimulation, however it remains unclear (45). Overall these symptoms resolved quickly after the sessions were completed. The increased sensitivity to the most common everyday life triggers of VID (e.g., being in supermarket or passenger in a car) was assessed by the VVAS questionnaire and it diminished in different conditions (see Table 2 for details).

**Table 2:** VVAS scores on the day prior to the treatment and on the last day of treatment (day 5).

**Questions VVAS**	**Pre**	**Day 5**	**Spearman's rank correlation coefficient**	***p*-value Spearman correlation**	**α-value (Holm–Bonferroni correction)**
(1) Walking through supermarket aisle	7	0.5	0.95	0.014	0.01
(2) Being a passenger in a car	6	0.4	0.81	0.05	0.012
3) Being under fluorescent lights	5	0.5	0.82	0.088	0.044
(4) Watching traffic at a busy intersection	4	0.3	**0.94**	**0.005**	**0.008**
(5) Walking through a shopping mall	7	0.5	0.87	0.054	0.018
6) Going down an escalator	4	0	0.71	0.182	0.182
7) Watching a movie at the movie theater	3	0	**0.98**	**0.005**	**0.008**
(8) Walking over a patterned floor	8	0.2	**0.99**	**0.001**	**0.006**
(9) Watching action television	6	0	**1**	**0.001**	**0.006**

*The Spearman's rank correlation coefficients and their p-values are showed in the fourth column. The significant p-values are highlighted when are lower than the respective alpha values (corrected using the Holm-Bonferroni correction procedure, last column)*.

The mean VVAS score was 5.6/10 during the pre-treatment assessment and reached an average of 0.26/10 the last day of treatment. When used a correlation analysis between the mean VVAS score against the 5 days of treatment a statistically significant correlation was reported (Spearman's rank correlation coefficient mean = −0.986; *p* < 0.001). More specifically, when examining each specific trigger, a statistically significant correlation was reported for four triggers (Table 2).

### Objective Outcome

**Figure 2:**
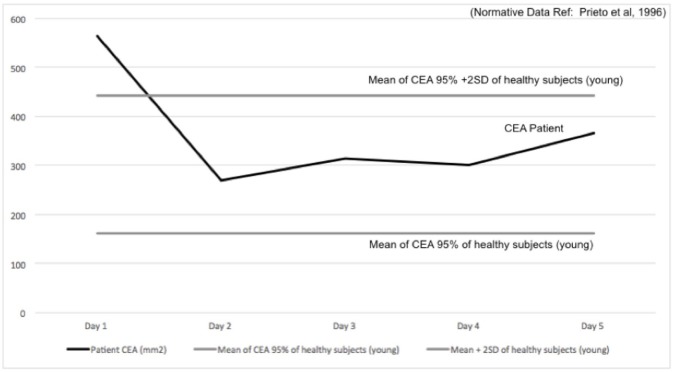
Confidence Ellipse Area (CEA, black line) in mm^2^ is reported over 5 days of treatment, plotted with the Normative Values (solid gray line: mean–dashed gray line: mean +2SD). CEA 95% confidence of healthy young subjects obtained from Prieto et al. (46).

The Confidence Ellipse Area (CEA) extracted from the posturography data identified abnormal results (mean CEA + two standard deviation) on the first OKN treatment day (46) (see Figure 2 for details). The CEA returned within the normative range on the pre-treatment measurement of day 2 and remained normal for all the following days *(Note: the player did not realize these improvements in postural stability)*.

### Follow Up—Return to sport

After the treatment, the player followed a comprehensive training programme (conditioning off/on ice, individually and with his team) and completed successfully the RTS protocol according to the RTS protocol of McCrory et al. (1, 12, 36). The player was able to play without symptoms in a competitive game (time on ice: 14 min) 15 days after the last session of combined VID and vestibular rehabilitation. Following this, he played regularly (time on ice on average: 18 min per game) without symptoms.

During questionnaire based follow up examination, which was performed 3 months after completion of the treatment the player reported no symptoms during all previously symptomatic conditions (SVQ mean score = 0.05; VVAS mean score = 0.11).

## Conclusion

The presented case report describes the presence of VID symptoms following a concussion in a professional ice hockey player with a history of multiple SRCs and of treated post-traumatic BPPV (following an initial concussion). It has been recently discovered that SRC are capable to lead to a disruption of central and peripheral vestibular pathways. The interaction of vestibular symptoms in concussed patients was here highlighted (47). During clinical examination it is essential to consider how central deficits may impede or alter a normal vestibular functioning in patients with refractory post-SRC dizziness, since this may lead to secondary symptoms such as VID (5). In this clinical case, VID symptoms could be identified and quantified through a series of functional examinations and standardized questionnaires for VID diagnosis in vestibular patients. By using a multidisciplinary approach, the player was successfully rehabilitated within 5 days of treatment over 2 weeks and successfully returned to play in 15 days after the completion of treatment. The treatment included a combination of OKN exposure (28) and VR. It was assumed, that the progressive exposure to OKN stimuli and the introduction of conflicting multi-sensory stimuli of increasing strength and complexity (visual, vestibular, and proprioceptive) to the player was able to successfully reduced his over reliance on visual input (26). This rehabilitation is believed to induce plastic, adaptive changes that decrease the magnitude of visual dependency (28). As reported in Figure 2, the players' postural sway before the treatment was larger than the normative value provided by the literature (46). As a result, in this case the combination of OKN with VR (ocular-motor and vestibular exercises) made the player overall more stable (Figure 2) and less sensitive to visual inputs (Table 2) (28, 48). The player's perception of improvement was not immediate and occurred only after the treatment was concluded. The player's improvement remained consistent after follow-up. This evident amelioration supports the need to further evaluate if the addition of OKN-based treatment consistently induces improvements in postural control in players who suffered SRC. On the base of this clinical case, we aim to raise awareness on the presence of VID symptom in concussed patients and on the potential use of OKN-treatment to reduce such symptoms. Moreover, this case would foster further research on the implementation of such technique as well as in the quantification of the plastic-adaptive processes of the use of OKN and VR in patients affected by SRC. One methodology to do so may be the use of advanced neuroimaging techniques such as functional magnetic resonance imaging (fMRI), which may help to better understand the pathophysiology of VID and the effects of interventions in concussed athletes.

## Data Availability Statement

The datasets generated for this study are available on request to the corresponding author.

## Ethics Statement

Ethical review and approval was not required for the study on human participants in accordance with the local legislation and institutional requirements. The patients/participants provided their written informed consent to participate in this study.

## Author Contributions

VM is the primary author and was involved in the creation of the protocol used to rehabilitate the player, she was involved in the data recording, analysis, and writing the manuscript. CM was involved in rehabilitating the patient. MB was involved in the rehabilitation program and follow up of the player. FR was involved in the data analysis and reviewing the manuscript. DA was involved in performing the patient vestibular test examination. AV reviewed the manuscript. GB was involved in reviewing the manuscript. NF-D diagnosed the patient, supervised the rehabilitation program, and reviewed the manuscript.

### Conflict of Interest

The authors declare that the research was conducted in the absence of any commercial or financial relationships that could be construed as a potential conflict of interest.
